# Causes of Death in People With Cardiovascular Disease: A UK Biobank Cohort Study

**DOI:** 10.1161/JAHA.121.023188

**Published:** 2021-11-06

**Authors:** Michael Drozd, Mar Pujades‐Rodriguez, Fei Sun, Kevin N. Franks, Patrick J. Lillie, Klaus K. Witte, Mark T. Kearney, Richard M. Cubbon

**Affiliations:** ^1^ Leeds Institute of Cardiovascular and Metabolic Medicine The University of Leeds Leeds UK; ^2^ Leeds Institute of Health Sciences School of Medicine The University of Leeds Leeds UK; ^3^ Leeds Cancer Centre St James’s Hospital Leeds Teaching Hospitals NHS Trust Leeds UK; ^4^ Department of Infection Castle Hill Hospital Hull University Hospitals NHS Trust Kingston Upon Hull UK; ^5^ Department of Cardiology Pneumonology, Angiology and Intensive Care Uniklinikum Aachen Aachen Germany

**Keywords:** cancer, cardiovascular, death, infection, Cardiovascular Disease, Mortality/Survival

## Abstract

**Background:**

Therapeutic advances have reduced cardiovascular death rates in people with cardiovascular diseases (CVD). We aimed to define the rates of cardiovascular and noncardiovascular death in people with specified CVDs or accruing cardiovascular multimorbidity.

**Methods and Results:**

We studied 493 280 UK residents enrolled in the UK Biobank cohort study. The proportion of deaths attributed to cardiovascular, cancer, infection, or other causes were calculated in groups defined by 9 distinct self‐reported CVDs at baseline, or by the number of these CVDs at baseline. Poisson regression analyses were then used to define adjusted incidence rate ratios for these causes of death, accounting for sociodemographic factors and comorbidity. Of 27 729 deaths, 20.4% were primarily attributed to CVD, 53.6% to cancer, 5.0% to infection, and 21.0% to other causes. As cardiovascular multimorbidity increased, the proportion of cardiovascular and infection‐related deaths was greater, contrasting with cancer and other deaths. Compared with people without CVD, those with 3 or more CVDs experienced adjusted incidence rate ratios of 7.0 (6.2–7.8) for cardiovascular death, 4.4 (3.4–5.6) for infection death, 1.5 (1.4–1.7) for cancer death, and 2.0 (1.7–2.4) for other causes of death. There was substantial heterogeneity in causes of death, both in terms of crude proportions and adjusted incidence rate ratios, among the 9 studied baseline CVDs.

**Conclusions:**

Noncardiovascular death is common in people with CVD, although its contribution varies widely between people with different CVDs. Holistic and personalized care are likely to be important tools for continuing to improve outcomes in people with CVD.

Age‐standardized cardiovascular disease (CVD) mortality rates have declined substantially over recent decades.^1^ Some evidence indicates this has been paralleled by an increasing proportion of noncardiovascular mortality in people with CVD. For example, noncardiovascular death now accounts for approximately 40% of deaths in people with chronic heart failure.^2^ However, the contemporary causes of death across a broad spectrum of CVDs, either alone or in combination, remain unclear, hindering the planning of strategies aiming to continue improving outcomes in people with CVD.

## Methods

### Study Design and Data Collection

The data that support the findings of this study are available from the corresponding author upon reasonable request. The UK Biobank study was a prospective observational study that recruited 502 505 residents in the United Kingdom aged 37 to 73 years between 2006 and 2010. This resource was developed with funding provided by the UK government and biomedical research charities with the aim to improve understanding of disease. All researchers are able to apply for access to this resource. Detailed information for study design and conduct are available at the UK Biobank website (https://www.ukbiobank.ac.uk). Participants attended 1 of 22 assessment centers across the United Kingdom. Biological measurements were recorded at baseline, and participants completed a touchscreen and nurse‐led interview, as previously described.^3^ UK Biobank received ethical approval from the National Health Service Research Ethics Service, and this analysis was undertaken under application number 59585. Participants provided written informed consent to participate in the UK Biobank study.

### Assessment of Demographic Factors and Morbidity

Age, sex, ethnicity, and socioeconomic status were collected at study recruitment by UK Biobank. Participants in UK Biobank were asked to define their own ethnicity (data‐field 21000) within the following major categories: "White," "Mixed," "Asian or Asian British," "Black or Black British," "Chinese," or "Other ethnic group"; in view of the small numbers of people in the non‐"White" groups, our analyses use pooled self‐defined ethnicity groups of "White" or "Non‐White". Self‐reported smoking status was recorded at recruitment defined as never, former, or current. We categorized the Townsend score collected at recruitment into quintiles, as previously described.^4^ Obesity was classified according to body mass index recorded at baseline based on the World Health Organization’s definitions: class 1 (30.0–34.9 kg/m²), class 2 (35.0–39.9 kg/m²), and class 3 (≥40 kg/m²). Medical conditions and operations were self‐reported at study recruitment during a face‐to‐face interview with a nurse. In addition to a broad range of cardiovascular diseases (defined in Table S1: abdominal aortic aneurysm, atrial fibrillation/flutter, coronary artery disease, heart failure, hypertension, peripheral vascular disease, stroke, valvular disease, venous thromboembolic disease), we also defined a broad range of other comorbidities based on our previously published work to incorporate this information into our models (respiratory disease, diabetes, cancer [previous or current], chronic liver disease, chronic kidney disease, other neurological disease [not stroke], psychiatric disorder, and chronic inflammatory and autoimmune rheumatic disease).^4^ The number of cardiovascular diseases (among the 9 listed) was also calculated for each participant as an index of cardiovascular multimorbidity. We excluded a total of 9225 (1.8%) participants because of missing baseline data or long‐term follow‐up data, or withdrawal of consent from the study. These included exclusions attributable to missing data for comorbidities (n=863), body mass index (n=3106), smoking (n=2949), ethnicity (n=2777), socioeconomic deprivation (n=624), and individuals lost to follow‐up or who withdrew consent (n=1314); some participants had >1 variable missing.

### Mortality Ascertainment

UK Biobank includes linked official national death registry data from National Health Service digital for participants in England and Wales, and from the National Health Service central register for participants in Scotland. In our analysis, we censored deaths to December 31, 2019 to ensure this was before the first recorded case of COVID‐19 in the United Kingdom.^5^ We extracted the underlying (primary) cause of death, coded according to the *International Classification of Diseases, Tenth Revision* (*ICD‐10*) from death certification data, classified as cardiovascular (I00–I99 [excluding infection codes: I32.0, I32.1, I33.0, I33.9, I38, I39, I40.0, I41.0–I41.2, I43.0, I52.0–I52.1, I68.1, I98.1]); cancer (C00–C97), infection (as we have previously defined),^4^ and any other remaining causes. We also conducted sensitivity analyses with alternative classification of cardiovascular death as I00 to I99 (without any exclusions) and infection codes (excluding any code beginning with the letter I).

### Statistical Analysis

Categorical variables were presented as number (percent). Adjusted cause‐specific mortality incidence rate ratios (IRRs) and their 95% CIs were estimated using Poisson regression models with exposure time modeled. Models were adjusted for all covariates including age, sex, socioeconomic deprivation (based on index of multiple deprivation quintile), smoking status, obesity, respiratory disease, diabetes, cancer (previous or current), liver disease, kidney disease, other neurological disease, psychiatric disorder, rheumatological disease, abdominal aortic aneurysm, atrial fibrillation/flutter, coronary artery disease, heart failure, hypertension, peripheral vascular disease, stroke, valvular disease, and venous thromboembolic disease. To assess the association of cardiovascular multimorbidity with cause of death, irrespective of the particular CVDs studied (abdominal aortic aneurysm, atrial fibrillation/flutter, coronary artery disease, heart failure, hypertension, peripheral vascular disease, stroke, valvular disease, venous thromboembolic disease), we categorized the number of baseline CVDs into 4 groups: none, 1, 2, and 3 or more cardiovascular conditions. Age was modeled using restricted cubic splines with 4 knots for cardiovascular death, cancer death, and other death, and 5 knots for infection death analyses, because these provided the best fit as assessed by the Akaike information and the Bayesian criterion (models including categorical, linear, or restricted cubic splines with 3, 4, and 5 knots and first‐degree and second‐degree fractional polynomials were compared). Crude mortality rates were calculated per 1000 person‐years follow‐up. All tests were 2‐sided, and statistical significance was defined as *P*<0.05. All statistical analyses used Stata/MP (version 16.1; StataCorp, College Station, TX).

## Results

Among 493 280 participants, 131 202 (26.6%) had 1 self‐reported CVD (within the 9 studied CVDs), 21 605 (4.4%) had 2 CVDs, and 3561 (0.7%) had 3 or more CVDs; characteristics of these groups are shown in Table 1. When defined by specified baseline CVD, there were 130 792 (26.5%) with hypertension, 22 847 (4.6%) with coronary artery disease, 12 386 (2.5%) with venous thromboembolic disease, 6996 (1.4%) with stroke, 4600 (0.9%) with valvular disease, 3649 (0.7%) with atrial fibrillation/flutter, 3160 (0.6%) with peripheral vascular disease, 781 (0.2%) with heart failure, and 418 (0.1%) with abdominal aortic aneurysm; characteristics of these groups are shown in Table 2.

**Table 1:** Characteristics of Participants According to Number of Baseline CVDs

	No CVD, n=336 912	One CVD, n=131 202	Two CVDs, n=21 605	Three or more CVDs, n=3561
Baseline age, y	56 (48–62)	61 (55–65)	63 (58–66)	64 (59–67)
Baseline age groups, y				
<45	44 403 (13.2%)	5862 (4.5%)	390 (1.8%)	44 (1.2%)
45 to <50	53 786 (16%)	10 116 (7.7%)	847 (3.9%)	110 (3.1%)
50 to <55	56 631 (16.8%)	16 294 (12.4%)	1788 (8.3%)	233 (6.5%)
55 to <60	61 412 (18.2%)	23 988 (18.3%)	3322 (15.4%)	537 (15.1%)
60 to <65	72 525 (21.5%)	38 989 (29.7%)	6763 (31.3%)	1091 (30.6%)
≥65	48 155 (14.3%)	35 953 (27.4%)	8495 (39.3%)	1546 (43.4%)
Age at death, y	69 (63–73)	71 (66–75)	71 (67–75)	71 (67–75)
Sex				
Men	142 366 (42.3%)	66 060 (50.3%)	13 587 (62.9%)	2416 (67.8%)
Women	194 546 (57.7%)	65 142 (49.7%)	8018 (37.1%)	1145 (32.2%)
Ethnicity^*^				
White	319 682 (94.9%)	123 581 (94.2%)	20 531 (95.0%)	3390 (95.2%)
Ethnic minority	17 230 (5.1%)	7621 (5.8%)	1074 (5.0%)	171 (4.8%)
SED quintile				
1	69 810 (20.7%)	24 958 (19.0%)	3443 (15.9%)	482 (13.5%)
2	68 585 (20.4%)	25 660 (19.6%)	3802 (17.6%)	573 (16.1%)
3	68 135 (20.2%)	25 853 (19.7%)	4088 (18.9%)	581 (16.3%)
4	67 224 (20%)	26 369 (20.1%)	4346 (20.1%)	715 (20.1%)
5	63 158 (18.7%)	28 362 (21.6%)	5926 (27.4%)	1210 (34.0%)
Smoking				
Never	192 620 (57.2%)	67 535 (51.5%)	8889 (41.1%)	1180 (33.1%)
Former	108 067 (32.1%)	50 990 (38.9%)	10 127 (46.9%)	1824 (51.2%)
Current	36 225 (10.8%)	12 677 (9.7%)	2589 (12.0%)	557 (15.6%)
Obesity				
Nonobese	273 984 (81.3%)	83 567 (63.7%)	12 118 (56.1%)	1828 (51.3%)
Class 1	48 006 (14.2%)	31 999 (24.4%)	6155 (28.5%)	1054 (29.6%)
Class 2	11 284 (3.3%)	10 936 (8.3%)	2250 (10.4%)	446 (12.5%)
Class 3	3638 (1.1%)	4700 (3.6%)	1082 (5.0%)	233 (6.5%)
Chronic respiratory disease	41 325 (12.3%)	18 136 (13.8%)	3691 (17.1%)	750 (21.1%)
Diabetes	6889 (2.0%)	13 129 (10.0%)	3859 (17.9%)	874 (24.5%)
Cancer	25 961 (7.7%)	12 389 (9.4%)	2229 (10.3%)	427 (12.0%)
Chronic liver disease	583 (0.2%)	294 (0.2%)	66 (0.3%)	13 (0.4%)
Chronic kidney disease	272 (0.1%)	696 (0.5%)	221 (1.0%)	82 (2.3%)
Neurological disease	4327 (1.3%)	1719 (1.3%)	397 (1.8%)	103 (2.9%)
Psychiatric disease	19 165 (5.7%)	8524 (6.5%)	1652 (7.6%)	306 (8.6%)
Rheumatological disease	6385 (1.9%)	3661 (2.8%)	864 (4%)	182 (5.1%)

CVD indicates cardiovascular disease; and SED, socioeconomic deprivation.

* Participants in UK Biobank were asked to define their own ethnicity (data‐field 21000) within the following major categories: "White," "Mixed," Asian or Asian British,' "Black or Black British,"' "Chinese," or "Other ethnic group"; in view of the small numbers of people in the non‐"White" groups, our analyses use pooled self‐defined ethnicity groups of "White" or "Non‐White".

**Table 2:** Characteristics of Participants According to Specified Baseline Cardiovascular Diseases

.	AAA, n=418	AF/flutter, n=3649	CAD, n=22 847	HF, n=781	Hypertension, n=130 792	PVD, n=3160	Stroke, n=6996	Valve, n=4600	VTE, n=12 386
Baseline age, y	65 (62–67)	63 (59–66)	63 (59–67)	61 (54–65)	61 (55–65)	61 (54–65)	62 (56–66)	61 (53–65)	61 (55–65)
Baseline age groups, yy									
<45	5 (1.2%)	61 (1.7%)	324 (1.4%)	35 (4.5%)	5020 (3.8%)	174 (5.5%)	215 (3.1%)	349 (7.6%)	601 (4.9%)
45 to <50	12 (2.9%)	142 (3.9%)	757 (3.3%)	69 (8.8%)	9124 (7.0%)	277 (8.8%)	393 (5.6%)	441 (9.6%)	951 (7.7%)
50 to <55	21 (5.0%)	254 (7%)	1682 (7.4%)	95 (12.2%)	15 456 (11.8%)	354 (11.2%)	708 (10.1%)	550 (12%)	1489 (12%)
55 to <60	40 (9.6%)	488 (13.4%)	3305 (14.5%)	144 (18.4%)	23 799 (18.2%)	535 (16.9%)	1140 (16.3%)	753 (16.4%)	2129 (17.2%)
60 to <65	110 (26.3%)	1213 (33.2%)	7270 (31.8%)	203 (26%)	39 427 (30.1%)	885 (28%)	2042 (29.2%)	1201 (26.1%)	3578 (28.9%)
≥65	230 (55.0%)	1491 (40.9%)	9509 (41.6%)	235 (30.1%)	37 966 (29%)	935 (29.6%)	2498 (35.7%)	1306 (28.4%)	3638 (29.4%)
Age at death, y	72 (69–75)	72 (68–75)	72 (68–75)	69 (64–73)	71 (66–75)	71 (66–75)	71 (66–75)	70 (66–74)	70 (66–74)
Sex									
Men	346 (82.8%)	2490 (68.2%)	16 299 (71.3%)	524 (67.1%)	67 996 (52%)	2007 (63.5%)	4070 (58.2%)	2078 (45.2%)	5016 (40.5%)
Women	72 (17.2%)	1159 (31.8%)	6548 (28.7%)	257 (32.9%)	62 796 (48%)	1153 (36.5%)	2926 (41.8%)	2522 (54.8%)	7370 (59.5%)
Ethnicity^*^									
White	404 (96.7%)	3601 (98.7%)	21 572 (94.4%)	747 (95.6%)	122 945 (94.0%)	3058 (96.8%)	6658 (95.2%)	4425 (96.2%)	11 901 (96.1%)
Ethnic minority	14 (3.3%)	48 (1.3%)	1275 (5.6%)	34 (4.4%)	7847 (6.0%)	102 (3.2%)	338 (4.8%)	175 (3.8%)	485 (3.9%)
SED quintile									
1	76 (18.2%)	762 (20.9%)	3592 (15.7%)	126 (16.1%)	24 128 (18.4%)	543 (17.2%)	1020 (14.6%)	908 (19.7%)	2190 (17.7%)
2	97 (23.2%)	788 (21.6%)	4010 (17.6%)	128 (16.4%)	25 112 (19.2%)	531 (16.8%)	1160 (16.6%)	914 (19.9%)	2316 (18.7%)
3	70 (16.7%)	753 (20.6%)	4227 (18.5%)	143 (18.3%)	25 598 (19.6%)	562 (17.8%)	1258 (18%)	874 (19%)	2368 (19.1%)
4	78 (18.7%)	724 (19.8%)	4574 (20%)	172 (22%)	26 277 (20.1%)	663 (21%)	1409 (20.1%)	913 (19.8%)	2507 (20.2%)
5	97 (23.2%)	622 (17%)	6444 (28.2%)	212 (27.1%)	29 677 (22.7%)	861 (27.2%)	2149 (30.7%)	991 (21.5%)	3005 (24.3%)
Smoking									
Never	92 (22.0%)	1799 (49.3%)	8379 (36.7%)	368 (47.1%)	65 795 (50.3%)	1116 (35.3%)	2852 (40.8%)	2444 (53.1%)	6166 (49.8%)
Former	247 (59.1%)	1624 (44.5%)	11 513 (50.4%)	328 (42%)	52 452 (40.1%)	1415 (44.8%)	3004 (42.9%)	1725 (37.5%)	4681 (37.8%)
Current	79 (18.9%)	226 (6.2%)	2955 (12.9%)	85 (10.9%)	12 545 (9.6%)	629 (19.9%)	1140 (16.3%)	431 (9.4%)	1539 (12.4%)
Obesity									
Nonobese	268 (64.1%)	2427 (66.5%)	13 664 (59.8%)	471 (60.3%)	78 985 (60.4%)	2224 (70.4%)	4427 (63.3%)	3465 (75.3%)	7624 (61.6%)
Class 1	114 (27.3%)	803 (22.0%)	6274 (27.5%)	180 (23.0%)	34 074 (26.1%)	663 (21%)	1733 (24.8%)	805 (17.5%)	2978 (24%)
Class 2	30 (7.2%)	275 (7.5%)	2067 (9.0%)	88 (11.3%)	12 205 (9.3%)	198 (6.3%)	595 (8.5%)	238 (5.2%)	1150 (9.3%)
Class 3	6 (1.4%)	144 (3.9%)	842 (3.7%)	42 (5.4%)	5528 (4.2%)	75 (2.4%)	241 (3.4%)	92 (2.0%)	634 (5.1%)
Chronic respiratory disease	70 (16.7%)	507 (13.9%)	3889 (17%)	135 (17.3%)	18 778 (14.4%)	484 (15.3%)	1170 (16.7%)	677 (14.7%)	2218 (17.9%)
Diabetes	53 (12.7%)	328 (9.0%)	4168 (18.2%)	107 (13.7%)	16 144 (12.3%)	411 (13%)	1011 (14.5%)	309 (6.7%)	1081 (8.7%)
Cancer	51 (12.2%)	363 (9.9%)	2145 (9.4%)	84 (10.8%)	12 288 (9.4%)	321 (10.2%)	748 (10.7%)	476 (10.3%)	1725 (13.9%)
Chronic liver disease	0 (0%)	11 (0.3%)	62 (0.3%)	2 (0.3%)	298 (0.2%)	10 (0.3%)	19 (0.3%)	20 (0.4%)	45 (0.4%)
Chronic kidney disease	6 (1.4%)	24 (0.7%)	181 (0.8%)	17 (2.2%)	929 (0.7%)	35 (1.1%)	68 (1.0%)	27 (0.6%)	108 (0.9%)
Neurological disease	8 (1.9%)	39 (1.1%)	395 (1.7%)	10 (1.3%)	1638 (1.3%)	60 (1.9%)	340 (4.9%)	78 (1.7%)	281 (2.3%)
Psychiatric disease	26 (6.2%)	167 (4.6%)	1637 (7.2%)	65 (8.3%)	8795 (6.7%)	216 (6.8%)	649 (9.3%)	311 (6.8%)	941 (7.6%)
Rheumatological disease	15 (3.6%)	115 (3.2%)	802 (3.5%)	27 (3.5%)	3840 (2.9%)	188 (5.9%)	269 (3.8%)	189 (4.1%)	531 (4.3%)

AAA indicates abdominal aortic aneurysm; AF, atrial fibrillation; CAD, coronary artery disease; HF, heart failure; PVD, peripheral vascular disease; SED, socioeconomic deprivation; valve, heart valve disease; and VTE, venous thromboembolism.

* Participants in UK Biobank were asked to define their own ethnicity (data‐field 21000) within the following major categories: "White," '"Mixed,"' "Asian or Asian British," "Black or Black British," "Chinese,"' or "Other ethnic group"; in view of the small numbers of people in the non‐"White" groups, our analyses use pooled self‐defined ethnicity groups of "White" or "Non‐White".

During a median follow‐up period of 10.9 years (interquartile range, 10.1–11.6 years) per participant, there were 27 729 deaths (censored December 31, 2019, before the COVID‐19 pandemic). Of these, 5648 (20.4%) were primarily attributed to CVD, 14 864 (53.6%) to cancer, 1385 (5.0%) to infection, and 5832 (21.0%) to other causes.

In participants with 1 CVD, only 22.4% of deaths were attributed to CVD, whereas 50.5% were attributed to cancer (data by cancer type are presented in Table S2), 5.7% to infection, and 21.4% to other causes (Figure A). As cardiovascular multimorbidity accrued, the proportion of cardiovascular and infection‐related deaths was higher, whereas cancer and other deaths were lower. In people with 3 or more CVDs, 43.1% of deaths were attributed to CVD; crude mortality rates are presented in Tables S3 and S4. Because the characteristics of people with accruing cardiovascular multimorbidity differ, we also examined the adjusted risk of cardiovascular, cancer, infection, or other death, relative to people without baseline CVD. As expected, the presence of 1 baseline CVD was associated with a higher risk of cardiovascular death, and a smaller increased risk of cancer or other death. Surprisingly, the risk of infection death was of a similar magnitude to cardiovascular death. In people with 3 or more CVDs (versus no CVD), the relative risk of cardiovascular death increased 7‐fold (IRR, 7.00; 6.24–7.84), followed by infection (IRR, 4.41; 3.44–5.64), other (IRR, 2.01; 1.72–2.35), and cancer (IRR, 1.52; 1.35–1.72) death. Notably, these estimates remained stable when assessed by the year of study recruitment, and differing durations of follow‐up (Tables S5 and S6). Sensitivity analyses using alternate definitions of infection and cardiovascular death, as described in the Methods section, revealed comparable findings (Tables S7 and S8). Hence, accruing cardiovascular multimorbidity is associated with an increasing contribution of cardiovascular and infection death, and higher adjusted risk of cardiovascular and infection death than cancer and other death.

**Figure 1:**
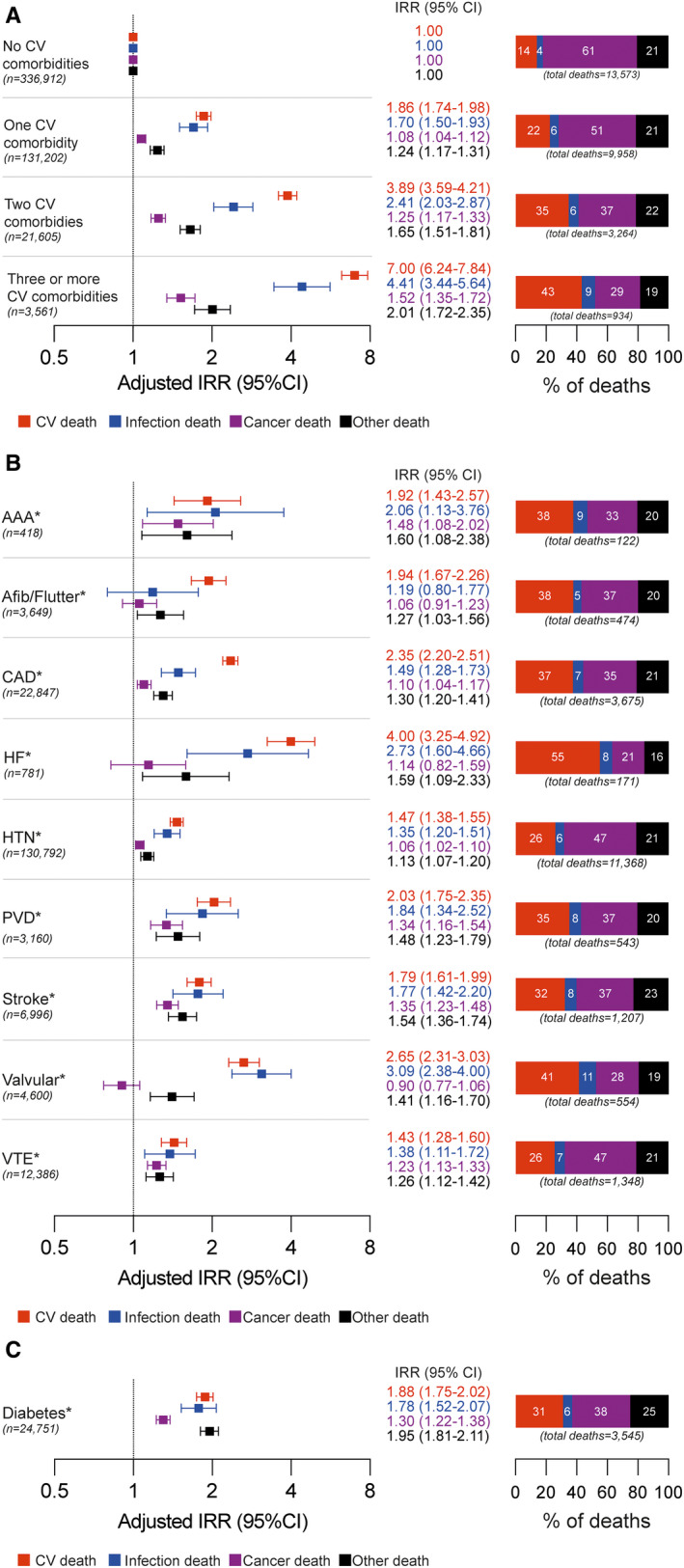
Causes of death according to baseline cardiovascular diseases (CVDs) and diabetes. Forest plots illustrate adjusted incidence rate ratios (IRRs) and 95% CIs (plotted on a log2 scale) for cardiovascular, infection, cancer, and other death according to number of baseline CVDs (**A**) or particular baseline CVDs (**B**) or diabetes (**C**) using multivariate Poisson regression analysis; bar charts illustrate the absolute percentage of deaths attributed to each cause. *In (**B)** and (**C)**, the reference group is people without the stated disease. AAA indicates abdominal aortic aneurysm; Afib, atrial fibrillation; CAD, coronary artery disease; CV, cardiovascular; flutter, atrial flutter; HF, heart failure; HTN, hypertension; PVD, peripheral vascular disease; and VTE, venous thromboembolism

Next, we explored how particular baseline CVDs were associated with cause of death (Figure B) and found substantial variation in the proportion of deaths attributed as cardiovascular, and the adjusted relative risk of cardiovascular death (versus people without that particular CVD), among the 9 studied CVDs. For example, cardiovascular death predominated in people with heart failure, whereas cancer death was most common in people with hypertension and venous thromboembolic disease. Similarly, the relative risk of cardiovascular death was much higher in people with heart failure (IRR, 4.00; 3.25–4.92), than hypertension (IRR, 1.47; 1.38–1.55), or venous thromboembolic disease (IRR, 1.43; 1.28–1.60). Despite the wider estimated confidence intervals in this analysis, it is apparent that the relative risk of infection death was also highest in people with heart failure (IRR, 2.73; 1.60–4.66) and valvular heart disease (IRR, 3.09; 2.38–4.00), and was elevated in people with all individual baseline CVDs except atrial fibrillation/flutter.

To provide broader context, we also assessed causes of death in people with diabetes (Figure C), an established risk factor for cardiovascular death. As expected, this was associated with a near 2‐fold increased risk of cardiovascular death (IRR, 1.88; 1.75–2.02), with 31% of all deaths being cardiovascular. The risk of infection and other death was also approximately doubled, but the confidence interval for cancer death was lower (IRR, 1.30; 1.22–1.38).

## Discussion

Our analyses show that attributed causes of death vary considerably across groups defined by baseline‐specific cardiovascular diseases. Unsurprisingly, rising baseline cardiovascular multimorbidity was associated with a greater proportion of cardiovascular deaths that persisted when assessed as adjusted rates. However, even in people with heart failure or multiple baseline cardiovascular diseases, approximately half of deaths were noncardiovascular, whereas this figure was approximately three‐quarters in people with hypertension or venous thromboembolic disease. As an absolute proportion, cancer was the largest contributor to noncardiovascular death, although in adjusted analyses, infection had the highest IRR of the noncardiovascular causes of death as baseline cardiovascular disease accrued.

These data have important clinical implications. First, cancer is a common cause of death in people with CVD, and lifestyle interventions for CVD might also reduce cancer risk, so this should be emphasized to patients.^6^ Second, the importance of cardio‐oncology is emphasized by the common occurrence of cancer death in people with CVD. Third, the increasing proportion of infection deaths, and the adjusted relative risk of infection death, in people with increasing cardiovascular multimorbidity suggests we need to promote established vaccinations and better understand and address mechanisms of infection risk in people with CVD.^4^ This is particularly important, because anti‐inflammatory therapies show promise in the management of cardiovascular disease, especially in light of signals for increased serious infection events in clinical trials of such approaches.^7^, ^8^

It is also important to acknowledge the limitations of our analysis. First, the UK Biobank data set is not representative of the wider UK population in terms of demography, ethnicity, socioeconomic deprivation, and disease prevalence^9^; hence, our findings should not be assumed as broadly generalizable. For example, the age range of the cohort may underrepresent cardiovascular and infection death and overrepresent cancer death in comparison with the general population.^10^ Second, we did not have data on incident cardiovascular disease during follow‐up, meaning that our data described associations with baseline disease status; hence, we may have underestimated the association between cardiovascular disease and subsequent death. Moreover, using self‐reported disease status to classify people as having cardiovascular disease may risk disease misclassification and reporting bias. Finally, our assessment of deprivation using the area‐based Townsend score is susceptible to misclassification of individuals because of changing deprivation area ranking over time, and the movement of individuals between areas. Moreover, the score assumes similar deprivation of all residents in a small geographical area, which might misclassify the status of some individuals.

In conclusion, noncardiovascular death is common in people with cardiovascular disease, although its proportional contribution varies widely between different cardiovascular diseases and according to the burden of cardiovascular multimorbidity. Holistic and personalized care are likely to be important tools for continuing to improve outcomes in people with CVD.

## Sources of Funding

This work was supported by the British Heart Foundation (FS/18/44/33792 and FS/12/80/29821).

## Disclosures

K.N.F. has received grants from Cancer Research UK and Yorkshire Cancer Research; he has also served as an independent contractor for AstraZeneca, Bristol‐Meyers Squibb, Roche Products Ltd, and Takeda Oncology. K.K.W. has served as an independent contractor for Abbott Vascular, AstraZeneca, Boehringer Ingelheim, Cardiac Dimensions, Medtronic, and Novartis; he has also received an unrestricted research grant from Medtronic. M.T.K. has received an unrestricted research grant from Medtronic. The remaining authors have no disclosures to report.

## Supporting information

Tables S1–S8
