# Supplementary material for: Causes of Death in People With Cardiovascular Disease: A UK Biobank Cohort Study
Source: J Am Heart Assoc. 2021 Nov 6;10(22):e023188. doi: 10.1161/JAHA.121.023188 (PMC8751922; doi:10.1161/JAHA.121.023188)
Supplement: Supplementary file 1 — Tables S1–S8 [file JAH3-10-e023188-s001.pdf]

SUPPLEMENTAL MATERIAL

Table S1. Cardiovascular disease definitions.

Disease	UK Biobank self-reported illnesses/operation included
Abdominal aortic aneurysm	Aortic aneurysm<br>Aortic aneurysm rupture<br>Aortic aneurysm/repair or stent
Atrial fibrillation/flutter	Atrial fibrillation<br>Atrial flutter
Coronary artery disease	Angina<br>Coronary angioplasty (PTCA) +/- stent<br>Coronary artery bypass grafts (CABG)<br>Heart attack/myocardial infarction<br>Triple heart bypass
Heart failure	Cardiomyopathy<br>Heart failure/pulmonary oedema<br>Hypertrophic cardiomyopathy (HCM / HOCM)
Hypertension	Essential hypertension<br>Hypertension
Peripheral vascular disease	Amputation of foot<br>Amputation of leg<br>Amputation of toe<br>Arterial embolism<br>Fem-pop bypass/leg artery bypass<br>Leg artery aneurysm repair<br>Leg artery angioplasty +/- stent<br>Leg claudication/ intermittent claudication<br>Other amputation<br>Peripheral vascular disease
Stroke	Brain haemorrhage<br>Ischaemic stroke<br>Stroke<br>Subarachnoid haemorrhage
Valvular disease	Aortic regurgitation / incompetence<br>Aortic stenosis<br>Aortic valve disease<br>Aortic valve repair/replacement<br>Heart valve problem/heart murmur<br>Heart valve surgery<br>Mitral regurgitation / incompetence<br>Mitral stenosis<br>Mitral valve disease<br>Mitral valve prolapse<br>Mitral valve repair/replacement<br>Other valve repair/replacement
Venous thromboembolic disease	Deep venous thrombosis (DVT)<br>Pulmonary embolism +/- DVT

All comorbidities are defined using self-reported illness/operation at verbal nurse led interview (UK Biobank data fields 20002 and 20004).

Diseases also adjusted for in modelling include: obesity (defined using BMI) and self-reported: chronic respiratory disease, diabetes, chronic liver disease, chronic kidney disease, other neurological disease, psychiatric disorder, and chronic inflammatory and autoimmune rheumatological disease as defined previously[2].

Table S2. Cancer deaths by sites.

	Cancer death					
**Cardiovascular comorbidity**	**Breast**	**Colorectal**	**Lung & bronchus**	**Prostate**	**Uterine**	**Other**
No CVD	820 (9.83%)	823 (9.87%)	1360 (16.31%)	441 (5.29%)	118 (1.42%)	4776 (57.28%)
One CVD	318 (6.32%)	469 (9.32%)	919 (18.27%)	324 (6.44%)	95 (1.89%)	2906 (57.76%)
Two CVDs	55 (4.51%)	90 (7.38%)	273 (22.38%)	83 (6.8%)	12 (0.98%)	707 (57.95%)
Three or more CVDs	4 (1.45%)	28 (10.18%)	69 (25.09%)	18 (6.55%)	3 (1.09%)	153 (55.64%)

CVD - cardiovascular disease

Table S3. Crude mortality rates per 1000 person-years of follow-up according to specific baseline cardiovascular comorbidities.

	CV death	Cancer death	Infection death	Other death	Total Deaths
**Abdominal aortic aneurysm**	11.66 (8.73-15.56)	10.14 (7.44-13.82)	2.79 (1.54-5.03)	6.34 (4.28-9.38)	30.92 (25.89-36.92)
**Atrial fibrillation/flutter**	4.74 (4.09-5.48)	4.68 (4.04-5.43)	0.67 (0.45-0.98)	2.53 (2.07-3.09)	12.61 (11.52-13.80)
**Coronary artery disease**	5.88 (5.58-6.20)	5.52 (5.22-5.83)	1.03 (0.90-1.16)	3.28 (3.05-3.52)	15.70 (15.20-16.22)
**Heart failure**	12.29 (10.04-15.04)	4.71 (3.39-6.52)	1.83 (1.08-3.09)	3.53 (2.42-5.15)	22.36 (19.24-25.97)
**Hypertension**	2.13 (2.05-2.21)	3.88 (3.77-3.98)	0.48 (0.45-0.52)	1.74 (1.67-1.81)	8.23 (8.08-8.38)
**Peripheral vascular disease**	5.89 (5.12-6.79)	6.17 (5.37-7.09)	1.27 (0.93-1.72)	3.43 (2.84-4.13)	16.76 (15.41-18.23)
**Stroke**	5.46 (4.94-6.03)	6.31 (5.76-6.93)	1.28 (1.04-1.57)	3.89 (3.45-4.37)	16.94 (16.01-17.92)
**Valvular disease**	4.78 (4.20-5.44)	3.24 (2.77-3.79)	1.29 (1.01-1.66)	2.26 (1.87-2.72)	11.57 (10.64-12.57)
**Venous thromboembolic disease**	2.66 (2.39-2.96)	4.87 (4.51-5.27)	0.68 (0.55-0.84)	2.21 (1.97-2.48)	10.43 (9.89-11.00)

Data are rates per 1000 person-years (95% CI). CV - cardiovascular

Table S4. Crude mortality rates per 1000 person-years of follow-up according to number of baseline cardiovascular comorbidities.

	CV death	Cancer death	Infection death	Other death	Total deaths
**No CVD**	0.52 (0.50-0.54)	2.30 (2.25-2.35)	0.15 (0.13-0.16)	0.78 (0.75-0.81)	3.74 (3.68-3.81)
**One CVD**	1.60 (1.53-1.67)	3.61 (3.52-3.72)	0.41 (0.38-0.44)	1.53 (1.47-1.60)	7.15 (7.01-7.30)
**Two CVDs**	5.11 (4.82-5.41)	5.49 (5.19-5.80)	0.93 (0.81-1.07)	3.16 (2.93-3.40)	14.68 (14.19-15.19)
**Three or more CVDs**	11.62 (10.53-12.81)	7.93 (7.04-8.92)	2.33 (1.88-2.90)	5.04 (4.35-5.85)	26.92 (25.25-28.70)

Data are rates per 1000 person-years (95% CI). CV – cardiovascular; CVD – cardiovascular disease

Table S5. Causes of death according to number of baseline cardiovascular comorbidities in each year of recruitment to UK Biobank.

	IRR (95% CI)							
	CV Death				Cancer death			
**Recruitment year**	**2006/2007** (n=52,627)	**2008** (n=184,267)	**2009** (n=170,328)	**2010** (n=86,058)	**2006/2007** (n=52,627)	**2008** (n=184,267)	**2009** (n=170,328)	**2010** (n=86,058)
**CV comorbidity (reference: none)**								
One CVD	1.87 (1.57-2.22)	1.92 (1.74-2.12)	1.91 (1.70-2.15)	1.54 (1.29-1.82)	1.08 (0.97-1.20)	1.07 (1.01-1.13)	1.10 (1.03-1.17)	1.04 (0.94-1.15)
Two CVDs	3.70 (3.00-4.57)	4.07 (3.61-4.60)	4.05 (3.50-4.70)	2.92 (2.33-3.66)	1.31 (1.12-1.54)	1.23 (1.11-1.36)	1.27 (1.13-1.42)	1.12 (0.94-1.34)
Three or more CVDs	6.87 (5.10-9.25)	6.60 (5.52-7.89)	7.56 (6.15-9.31)	6.85 (5.05-9.29)	1.58 (1.16-2.17)	1.59 (1.32-1.92)	1.36 (1.08-1.71)	1.51 (1.07-2.14)

	IRR (95% CI)							
	Infection death				Other death			
**Recruitment year**	**2006/2007** (n=52,627)	**2008** (n=184,267)	**2009** (n=170,328)	**2010** (n=86,058)	**2006/2007** (n=52,627)	**2008** (n=184,267)	**2009** (n=170,328)	**2010** (n=86,058)
**CV comorbidity (reference: none)**								
One CVD	1.88 (1.37-2.58)	1.72 (1.43-2.08)	1.46 (1.16-1.84)	1.91 (1.34-2.71)	1.24 (1.06-1.45)	1.23 (1.12-1.35)	1.23 (1.11-1.37)	1.24 (1.06-1.47)
Two CVDs	2.69 (1.77-4.08)	2.38 (1.83-3.10)	2.04 (1.47-2.82)	2.79 (1.71-4.56)	1.36 (1.07-1.73)	1.77 (1.55-2.03)	1.57 (1.34-1.85)	1.71 (1.33-2.20)
Three or more CVDs	3.86 (2.06-7.22)	5.51 (3.88-7.83)	2.90 (1.76-4.80)	4.49 (2.09-9.64)	1.65 (1.09-2.51)	2.09 (1.64-2.66)	2.05 (1.55-2.70)	1.90 (1.17-3.09)

2006/2007 merged due to small number (n=3,675) of participants recruited in 2006.

*Fully adjusted models include age (modelled by use of restricted cubic splines with five knots for infection death, four knots for all other analyses), sex, socioeconomic deprivation, ethnicity, smoking, obesity, respiratory disease, diabetes, cancer, liver disease, kidney disease, neurological disease, psychiatric disease, rheumatological disease. CV – cardiovascular; CVD – cardiovascular disease.

Table S6. Causes of death according to number of baseline cardiovascular comorbidities after 5- or 9-years of follow-up.

	IRR (95% CI)							
	CV Death		Cancer Death		Infection Death		Other death	
**Timepoint**	**5 years**	**9 years**	**5 years**	**9 years**	**5 years**	**9 years**	**5 years**	**9 years**
**CV comorbidity (reference: none)**								
One CVD	1.66 (1.48-1.87)	1.90 (1.76-2.05)	1.08 (1.02-1.15)	1.07 (1.02-1.11)	1.98 (1.51-2.60)	1.65 (1.41-1.92)	1.17 (1.03-1.33)	1.24 (1.16-1.34)
Two CVDs	3.95 (3.43-4.54)	4.08 (3.71-4.48)	1.28 (1.15-1.42)	1.18 (1.10-1.27)	2.37 (1.58-3.56)	2.55 (2.07-3.14)	1.68 (1.41-2.02)	1.67 (1.50-1.86)
Three or more CVDs	6.76 (5.55-8.23)	7.01 (6.15-8.00)	1.55 (1.27-1.90)	1.40 (1.22-1.61)	7.83 (4.91-12.48)	4.56 (3.41-6.10)	2.20 (1.64-2.95)	1.84 (1.52-2.23)

*Fully adjusted models include age (modelled by use of restricted cubic splines with five knots for infection death, four knots for all other analyses), sex, socioeconomic deprivation, ethnicity, smoking, obesity, respiratory disease, diabetes, cancer, liver disease, kidney disease, neurological disease, psychiatric disease, rheumatological disease. CV – cardiovascular; CVD – cardiovascular disease.

Table S7. Cardiovascular and infection death classification sensitivity analyses - Causes of death according to number of baseline cardiovascular comorbidities.

Total of 61 death events occurred that were reclassified for alternative classification.

	IRR (95% CI)			
	CV death		Infection death	
**Classification**	**Presented**	**Alternative**	**Presented**	**Alternative**
One CVD	1.86 (1.74-1.98)	1.87 (1.75-1.99)	1.70 (1.50-1.93)	1.67 (1.47-1.90)
Two CVDs	3.89 (3.59-4.21)	3.92 (3.62-4.24)	2.41 (2.03-2.87)	2.26 (1.89-2.70)
Three or more CVDs	7.00 (6.24-7.84)	7.15 (6.40-8.00)	4.41 (3.44-5.64)	3.85 (2.97-5.00)

*Fully adjusted models include age (modelled by use of restricted cubic splines with five knots for infection death, four knots for all other analyses), sex, socioeconomic deprivation, ethnicity, smoking, obesity, respiratory disease, diabetes, cancer, liver disease, kidney disease, neurological disease, psychiatric disease, rheumatological disease. CV – cardiovascular; CVD – cardiovascular disease.

Table S8. Cardiovascular and infection death classification sensitivity analyses - Causes of death according to specific baseline cardiovascular comorbidities.

Total of 61 death events occurred that were reclassified for alternative classification.

	IRR (95% CI)			
	CV death		Infection death	
Classification	Presented	Alternative	Presented	Alternative
Abdominal aortic aneurysm	1.92 (1.43-2.57)	1.88 (1.40-2.52)	2.06 (1.13-3.76)	2.24 (1.23-4.09)
Atrial fibrillation/flutter	1.94 (1.67-2.26)	1.92 (1.65-2.24)	1.19 (0.80-1.77)	1.2 (0.79-1.81)
Coronary artery disease	2.35 (2.2-2.51)	2.35 (2.20-2.51)	1.49 (1.28-1.73)	1.44 (1.23-1.68)
Heart failure	4.00 (3.25-4.92)	4.06 (3.31-4.98)	2.73 (1.60-4.66)	2.32 (1.27-4.22)
Hypertension	1.47 (1.38-1.55)	1.46 (1.38-1.55)	1.35 (1.20-1.51)	1.34 (1.20-1.51)
Peripheral vascular disease	2.03 (1.75-2.35)	2.02 (1.75-2.34)	1.84 (1.34-2.52)	1.88 (1.37-2.59)
Stroke	1.79 (1.61-1.99)	1.80 (1.62-2.00)	1.77 (1.42-2.2)	1.70 (1.35-2.13)
Valvular disease	2.65 (2.31-3.03)	2.79 (2.45-3.18)	3.09 (2.38-4.00)	2.45 (1.83-3.30)
Venous thromboembolic disease	1.43 (1.28-1.60)	1.44 (1.29-1.61)	1.38 (1.11-1.72)	1.35 (1.08-1.70)

*Fully adjusted models include age (modelled by use of restricted cubic splines with five knots for infection death, four knots for all other analyses), sex, socioeconomic deprivation, ethnicity, smoking, obesity, respiratory disease, diabetes, cancer, liver disease, kidney disease, neurological disease, psychiatric disease, rheumatological disease, abdominal aortic aneurysm, atrial fibrillation/flutter, coronary artery disease, heart failure, hypertension, peripheral vascular disease, stroke, valvular disease, venous thromboembolic disease.
